# Research on an Improved YOLOv8 Detection Method for Surface Defects of Optical Components

**DOI:** 10.3390/mi16121373

**Published:** 2025-12-01

**Authors:** Bei Ma, Jialong Zhao, Shun Zhou, Hongjun Wang, Junqi Xu, Bingcai Liu, Jingyao Hou, Weiguo Liu

**Affiliations:** School of Optoelectronic Engineering, Xi’an Technological University, Xi’an 710021, China; fazghch@126.com (B.M.); 17392085368@163.com (J.Z.); zsemail@126.com (S.Z.); whj0253@sina.com (H.W.); jqxu2210@163.com (J.X.); liubingcai@xatu.edu.cn (B.L.); 18392385119@163.com (J.H.)

**Keywords:** optical components, surface defects, attention mechanism, deep learning

## Abstract

Optical components are extensively used in aerospace, microelectronic equipment, precision optical measurement, laser optics and other fields. Surface defects on optical components can significantly impact system performance, necessitating specialized detection methods. However, technical challenges persist in achieving high-resolution, high-precision and efficient optical surface defect detection. To address this, we propose an improved YOLOv8-based object recognition algorithm. By incorporating the BRA attention mechanism into YOLOv8’s backbone network, multi-scale feature maps are processed to enhance adaptability to complex scenarios. Simultaneously, replacing the feature fusion module with the Context-GuideFPN module enables contextual guidance and adaptive adjustments during multi-scale feature integration without excessive computational overhead. Experimental results on our high-quality microscopic dark-field image dataset demonstrate that the enhanced BACG-YOLOv8 achieves excellent performance in optical component defect detection. The optimized network accurately extracts defect details, particularly demonstrating refined edge feature extraction while effectively suppressing noise interference. This significantly reduces detection errors and improves defect extraction accuracy.

## 1. Introduction

As the core components of modern optical systems, the surface quality of optical elements directly determines system performance parameters, including image clarity, energy transfer efficiency and laser damage threshold [1]. Surface defects such as scratches, pitting, bubbles and edge cracks can induce light scattering, resulting in degraded beam quality and increased stray light [2,3]. In high-power laser systems, these defects may even cause catastrophic damage. Table 1 lists typical defect types. High-precision and efficient surface defect detection serves as a critical component in precision manufacturing and quality control processes.

Optical defect detection methods can be broadly categorized into conventional inspection techniques, advanced light scattering-based approaches and modern AI-driven solutions. In conventional inspection technologies, visual inspection follows the MIL-PRF-13830B standard, relying on operator experience for macroscopic defect screening using a 10× stereo microscope in dark-field environments (Darkfield Microscope Imaging System SPZF0763+ZF-1.42C, Guilin Vision Encyclopedia Optoelectronics Technology Co., Ltd., Guilin, China). Interferometric measurement utilizes wavelength-shifted coherent interferometry (PSI) with a 632.8 nm excitation light source for nanoscale surface shape and roughness analysis (RMS precision 0.1 nm), though it exhibits limited sensitivity to localized micro-defects. Advanced light scattering-based methods employ polarization BRDF technology, which distinguishes pits, subsurface damage and microroughness by analyzing the polarization state (ss/sp/pp/ps) and angular distribution of scattered light [4]. Simulation and experimental results demonstrate that BRDF_pp_ exhibits the highest sensitivity to scattering angle variations. Angle-resolved scattering (ARS) measures spatially distributed scattered light (θ = 5–85°), while the combination of bidirectional scattering distribution function (BSDF) models enables inversion of subsurface damage parameters with angular resolution ≤0.1°. In AI-powered machine vision and intelligent inspection systems, dark-field scattering imaging enhances the signal-to-noise ratio by projecting defects as bright images against a dark background (e.g., the ARGOS system employs line scan cameras with rotating stages) [5]. The multi-angle ring light source eliminates illumination blind spots while accommodating curved component detection. The deep learning framework utilizes U-Net for defect segmentation, ResNet for classification and YOLOv5 for real-time localization, achieving over 99% detection accuracy (for Class 5/1 0.16 defects) [6]. Detailed comparisons are provided in Table 2.

The detection technology for optical component surface defects is rapidly evolving from traditional manual visual inspection and single-method approaches to high-precision optical scanning, intelligent systems and multimodal integration [7]. Currently, dark-field imaging based on scattering theory and deep learning-based AOI form the dual pillars of research and application, while cutting-edge techniques such as deep ultraviolet interferometry and confocal 3D reconstruction play pivotal roles in advanced applications. Looking ahead, overcoming precision and efficiency bottlenecks, adapting to complex curved surfaces, advancing AI applications and promoting standardization and domestic production will be the core drivers for the field’s sustained development [5,8,9].

With continuous breakthroughs in artificial intelligence technology, deep learning has shown significant advantages in various fields. Its automated feature recognition and large-scale data processing capabilities have made it a focal point in defect detection on complex surfaces. Compared to traditional methods, deep learning can identify intricate features more rapidly and accurately, providing industrial production with more efficient and reliable solutions [10]. This research utilizes a microscopic dark-field imaging system to capture surface defects on optical components and constructs a dark-field defect dataset. By integrating the YOLOv8 network for training on this dataset, we ultimately achieve accurate and efficient defect extraction.

## 2. YOLOv8 Network

In industrial inspection, balancing the accuracy and speed of the model is essential. However, current inspection methods face challenges such as large model size and low accuracy, particularly under low-light conditions. YOLOv8 is a deep learning network proposed by the Ultralyticsgongs team, which integrates multiple tasks including object detection, instance segmentation and real-time tracking. Compared to previous YOLO series algorithms, YOLOv8’s primary advancement lies in adopting the Anchor-Free detection method, a center detection algorithm without anchor boxes. This method decouples the head structure, separating classification and regression to avoid interference caused by anchor box selection (quantity and size). YOLOv8 employs Decoupled-Head as its final detection head, significantly improving network performance. Additionally, the network replaces the C3 module with the C2f module, which enhances gradient flow while maintaining a lightweight design, further optimizing the network structure. YOLOv8 provides five models of varying sizes. To balance the accuracy and speed of detection, the YOLOv8 is selected as the benchmark for optical component defect detection. It is an efficient real-time object detection algorithm with exceptional performance and speed [11].

### YOLOv8 Network Structure

**Figure 1:**
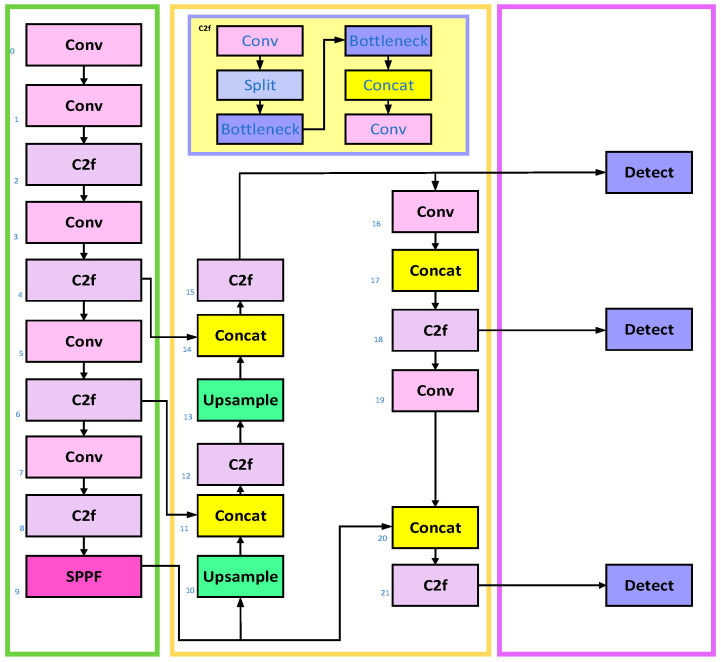
YOLOv8 network structure.

YOLOv8 is mainly divided into four parts: input, backbone, neck river and output [6,12,13]. Taking YOLOv8n as an example, its network structure is shown in Figure 1.

The backbone network serves as the foundation of YOLOv8 [14], responsible for extracting features from input images. This component employs the C2f module as its core building block, which demonstrates fewer parameters and enhanced feature extraction capabilities. Through optimized structural design, the C2f module reduces redundant parameters and improves computational efficiency. Additionally, the backbone integrates techniques such as deep separable convolution and dilated convolution to further enhance feature extraction performance. The neck network in YOLOv8 handles multi-scale feature fusion. It includes the SPPF module (Spatial Pyramid Pooling Fast), which performs multi-scale pooling operations by stitching feature maps across different scales to improve detection accuracy for objects of varying sizes. It also incorporates the PAA module (Probabilistic Anchor Assignment) and two PAN modules (Path Aggregation Network), which aggregate features across hierarchical levels to enhance feature map expressiveness through top-down and bottom-up path propagation. The head network, the critical component for final detection and classification tasks in YOLOv8, consists of a detection head and a classification head. The detection head predicts bounding box regression values and object confidence levels for each anchor box, while the classification head performs global average pooling on feature maps to classify objects, generating probability distributions for each category. The architecture of YOLOv8’s head module is specifically designed to handle multi-category classification tasks efficiently. This network design delivers an outstanding performance in object recognition, particularly when processing targets of varying sizes, as it provides richer and more accurate feature representations. Furthermore, the model incorporates optimization techniques such as the Anchor-Free mechanism and adaptive NMS (Non-Maximum Suppression with Non-Local Spatial Aggregation), which significantly improve detection accuracy and computational efficiency.

## 3. The Improved BACG-YOLOv8 Network Model Is Designed

Considering the characteristics of the optical component surface defect dataset, such as its dense target distribution, diverse size ranges and complex background, as well as the problems of missed detections, low accuracy and large parameter size in the baseline YOLOv8 [15], this paper proposes the following improvements. The backbone module of BACG-YOLOv8 incorporates the BRA attention mechanism to address feature maps of different scales. The original feature fusion layer is replaced with a content-guided feature fusion layer, and the SE attention mechanism is introduced.

### 3.1. BRA Attention Mechanism

The BRA attention mechanism achieves dynamic sparsity through a dual-layer routing strategy, prioritizing key-value pairs most relevant to queries. This approach reduces computational costs and enhances computational efficiency [16]. It is applicable to various visual tasks, particularly in the BiFormer architecture, where adaptive attention to critical markers boosts performance in small object detection.

**Figure 2:**
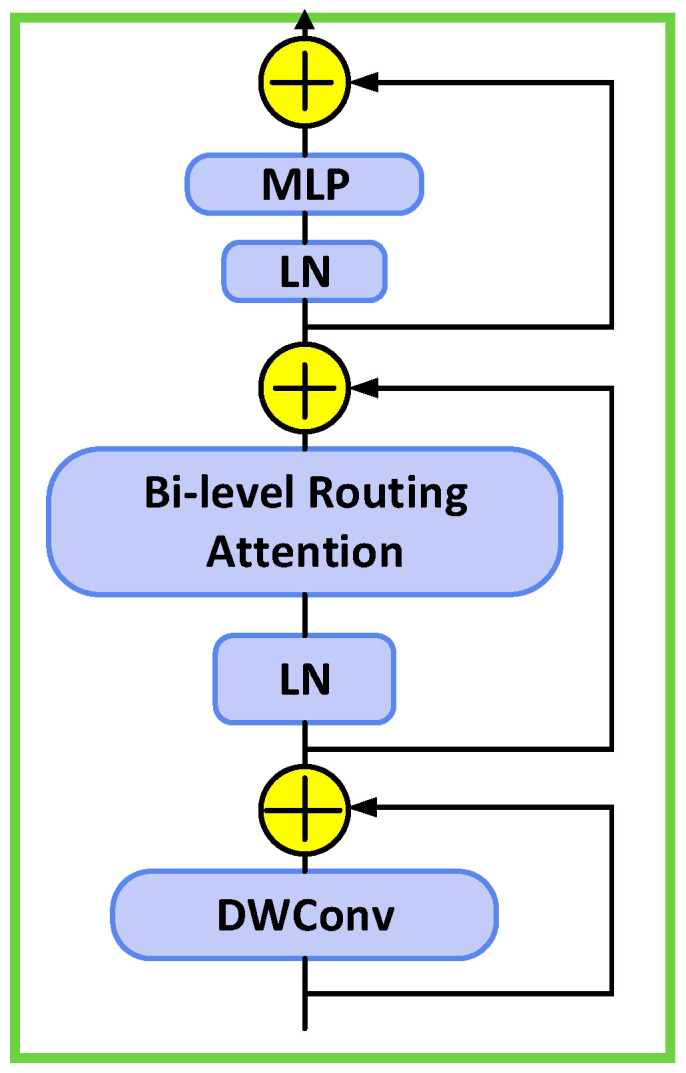
BiFormer module diagram.

BiFormer adopts a four-stage pyramid structure, with each stage incorporating distinct BRA modules and multi-layer perceptron (MLP) modules for feature transformation. The model adjusts the regional partition factor *S* and the number of regions to focus on depending on the task, to achieve optimal computational efficiency. Figure 2 shows the module diagram of BiFormer.

### 3.2. Context-GuideFPN Module

#### 3.2.1. Introduce the SE Attention Mechanism

**Figure 3:**
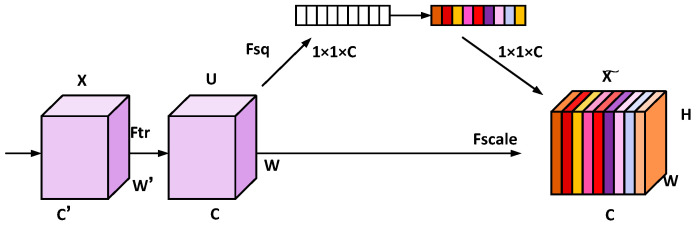
Basic structure diagram of the SE module.

In the defect detection of optical components, noise can interfere with the imaging quality, mask the true defect signals and reduce detection accuracy. It not only may cause the omission of minor defects but also trigger false defect misjudgments. which seriously affects the reliability of detection. Therefore, effective noise reduction techniques must be adopted. The Squeeze-and-Excitation (SE) attention mechanism enhances feature representation in convolutional neural networks (CNNs) by explicitly modeling inter-channel dependencies and adaptively recalibrating channel responses. This mechanism improves the network’s sensitivity to informative features while suppressing irrelevant ones. In the YOLOv8 architecture, the SE block significantly boosts performance compared to state-of-the-art CNNs by introducing cross-channel attention. The integration of this module achieves minimal computational overhead, maintaining a lightweight design without substantially increasing model complexity or computational load in the Figure 3.

#### 3.2.2. The Context-GuideFPN Module Is Introduced

**Figure 4:**
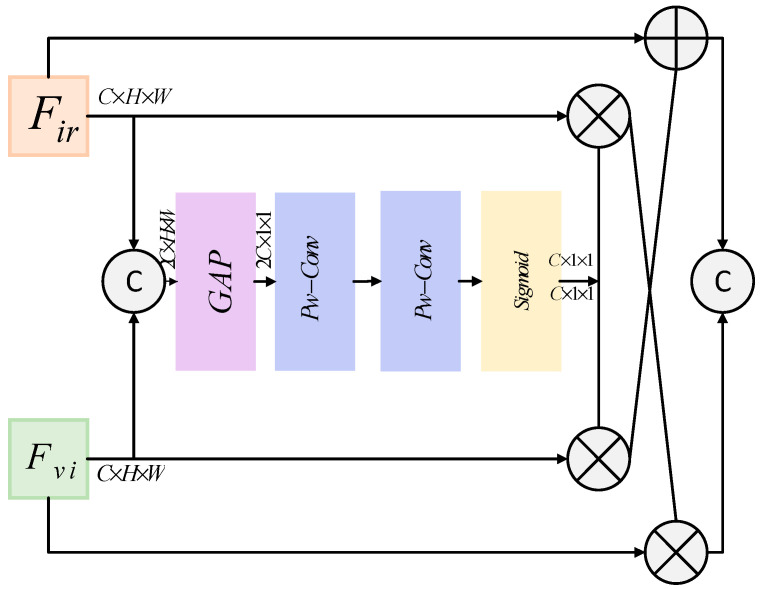
Surface detail fusion module based on channel-space attention mechanism.

The Superficial Detail Fusion Module (SDFM), a pivotal component in the PSFusion network, serves to process and fuse shallow-level features from multimodal images. These features are rich in fine-grained details and structural information, which are critical for generating high-quality fused images in the Figure 4. The SDFM excels in comprehensively extracting and leveraging structural and detailed information from multimodal sources, achieving enhanced fusion outcomes with greater fidelity and precision.

**Figure 5:**
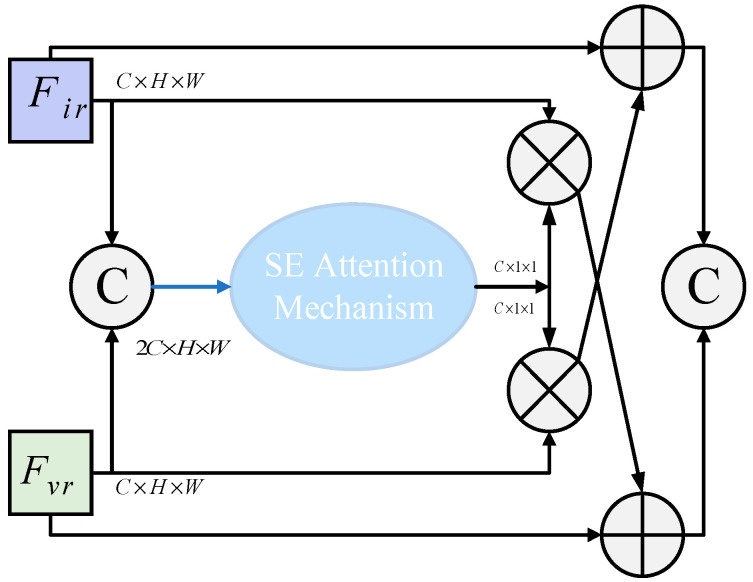
Improved Context-GuideFPN Module.

Referring to the above structure and by comparing it with the architecture diagram of the YOLOv8 model, the improved module is designed as shown in Figure 5. While maintaining equal channel numbers, the system first connects inputs via the channels, replaces the original SDFM with an SE attention mechanism, and extracts 2*C* × *H* × *W* from the connection points. This process generates adaptive weights for final concatenation operations. The modified network structure, based on the classic YOLOv8 model architecture, is illustrated in the diagram below.

To capture image features effectively at different levels, the module constructed in this paper takes into account the guidance and adaptive adjustment of context information during the multi-scale feature fusion process. This module integrates contextual guidance and adaptive adjustments through multi-scale feature fusion. By leveraging the SE attention mechanism, it captures and utilizes critical contextual information during feature integration, thereby enhancing feature representation effectiveness and guiding the model to better identify target information, which improves recognition accuracy. The weighted feature reorganization operation strengthens key features while suppressing irrelevant ones, enhancing the discriminative power of feature maps. As shown in Figure 5, these enhancements are relatively straightforward to implement while introducing minimal computational overhead.

## 4. Experiment and Analysis

### 4.1. Surface Defect Characteristic Dataset and Platform Construction

#### 4.1.1. Defect Image Acquisition Based on Microscopic Dark-Field Imaging

**Figure 6:**
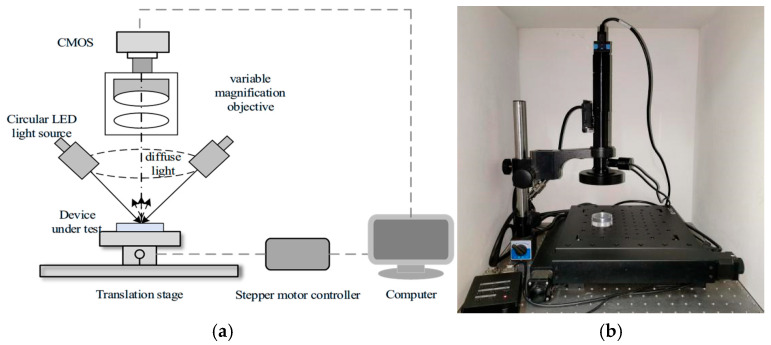
Schematic diagram of the composition of the microscopic dark-field imaging system. (**a**) Schematic diagram of dark-field imaging system. (**b**) Photo of the physical device for dark-field imaging.

According to the basic principle of microscopic dark-field imaging, a dark-field imaging system is constructed for the acquisition of images of surface defects on optical components. The system is primarily composed of an image acquisition module and a two-dimensional translation stage control module. The structural schematic diagram and physical image are shown in Figure 6.

The image acquisition module features a tilted illumination structure consisting of a CMOS sensor, zoom microscope objective lens and LED ring light source. Below the imaging system is the two-dimensional translation stage control module, primarily composed of a two-dimensional translation stage and a stepper motor controller, designed to move optical components under test. A data interface connects the CMOS camera to the computer, enabling real-time transmission of defect images captured on optical surfaces. The zoom controller ensures seamless zoom functionality with precise magnification control, while the ring light source regulator manages illumination through the LED ring light. Specific parameters of the zoom microscope objective lens are detailed in Table 3 [17].

**Figure 7:**
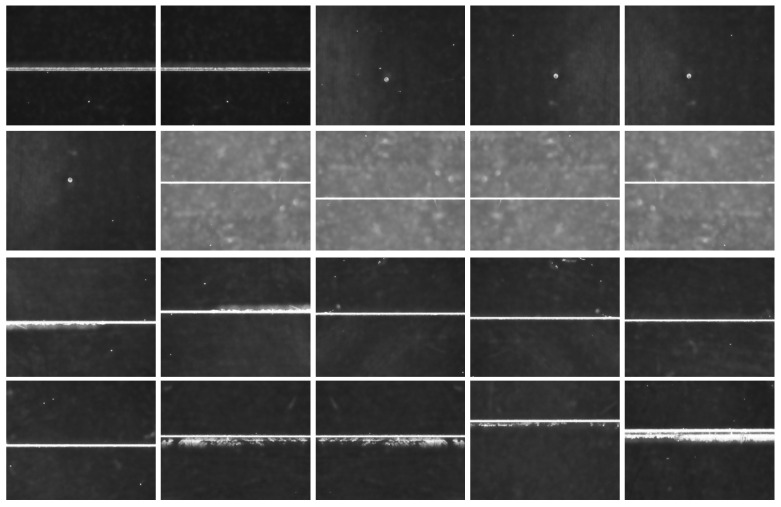
Micro dark-field imaging of surface defects of optical components.

The above microscopic dark-field imaging system was employed to capture surface defect images of optical components. Dark-field imaging technology effectively enhances the visibility of micro-scale defects, facilitating precise identification and analysis. A total of 450 dark-field images of surface defects on optical components were collected, with representative examples shown in Figure 7.

#### 4.1.2. Surface Defect Image Preprocessing

**Figure 8:**
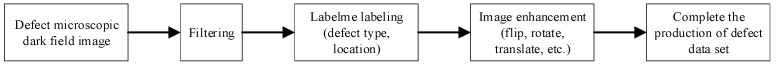
Flow chart of micro dark-field surface defect dataset production.

In the development of deep learning network models, this study employs defect feature images captured by a microscopic dark-field imaging system as the training dataset. To better align the defect images with the model’s requirements, the images undergo preprocessing and enhancement. As illustrated in Figure 8, the complete workflow for creating a dark-field surface defect image dataset comprises three key phases: image preprocessing, annotation and data augmentation.

(1) Image filter processing

To ensure convolutional neural networks accurately extract defect features, high-quality image data is essential. Noise contamination in captured defect images may lead to biased feature extraction, adversely affecting detection accuracy. Therefore, smoothing processing of acquired defect images is necessary. Considering the characteristics of microscopic dark-field defect images, the noise reduction process should suppress interference from non-defective regions while enhancing contrast in defect-affected areas. Common image filtering methods include Gaussian filtering, mean filtering and median filtering.

**Figure 9:**
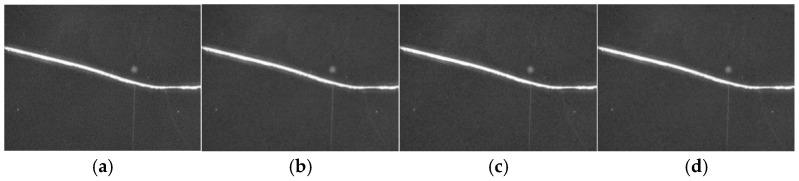
Results of different filtering methods. (**a**) original image, (**b**) mean filter, (**c**) median filtering, and (**d**) Gaussian filtering.

Figure 9 demonstrates the results of different filtering techniques. Compared with the original dark-field defect image input, all three filtering methods effectively suppress noise. However, both mean and Gaussian filtering produce blurred defect edges, which may compromise subsequent feature extraction. In contrast, median filtering reduces background noise while preserving edge details. Therefore, median filtering was selected as the preprocessing algorithm, providing a high-quality foundation for subsequent defect size measurement.

#### 4.1.3. Image Annotation and Enhancement of Surface Defects

(1) Defect image annotation

Optical components may exhibit non-target defects such as dust or bubbles on their surfaces. Therefore, it is crucial to annotate defect regions in filtered images by clearly distinguishing between defective and non-defective areas. Specifically, the scratches and pitting to be inspected should be confined to the region of interest (ROI), while other impurities in the image are excluded from this area for temporary exclusion. This annotation serves as a reference for model training, enabling precise defect recognition and localization. During network training, the original image acts as input data, while the corresponding label data facilitates loss function calculation, thereby enabling backpropagation optimization of the neural network.

**Figure 10:**
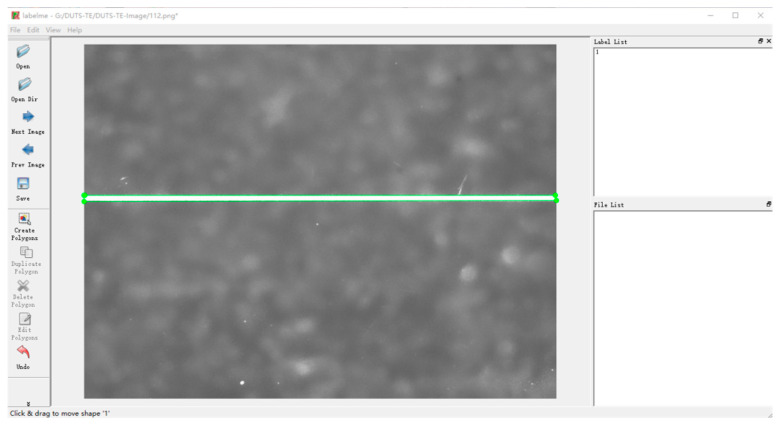
Defect annotation interface in the Labelme.

To extract target defect regions from captured images and generate corresponding label maps, this paper utilizes the open-source annotation tool Labelme (version 4.5.13) for marking surface defects on optical components under test. The tool supports batch annotation processing, allowing users to import defect image folders for automatic labeling. The specific annotation process involves the following: using point annotation lines to outline defect edges in imported images, applying appropriate labels to generated shape regions and saving the annotated files. Figure 10 illustrates the Labelme defect annotation interface.

**Figure 11:**
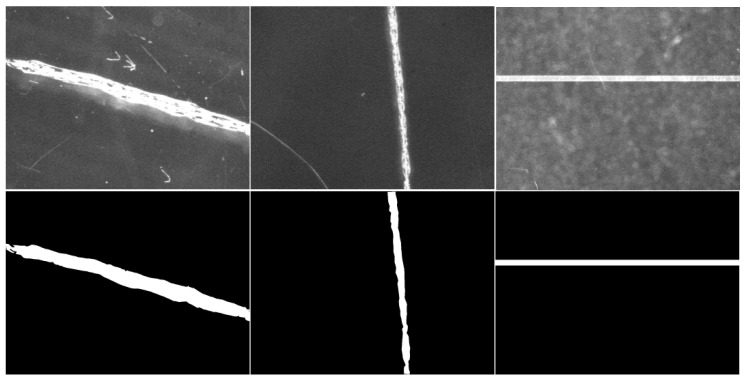
Original defect image and corresponding label image.

After using the Labelme tool to annotate target defect areas, a JSON file is generated. By parsing this JSON file with Python, the defect label images can be obtained that correspond to the original defect images. As shown in Figure 11, the defect images and their corresponding label images are displayed here. The defect label images visually highlight the detailed features of defects, providing reliable data to support model training and evaluation.

(2) Defect sample expansion

During deep learning network training, sufficient training samples are essential to prevent overfitting and under fitting, ensuring model convergence and achieving higher detection rates in testing. The optical component surface defect dataset developed in this study contains 450 defect images, representing a small-sample dataset. To enhance the model’s detection capability and generalization ability, data augmentation techniques were applied to the original dataset through transformations such as rotation, translation and flipping, thereby increasing the sample quantity.

a Image flipped

**Figure 12:**
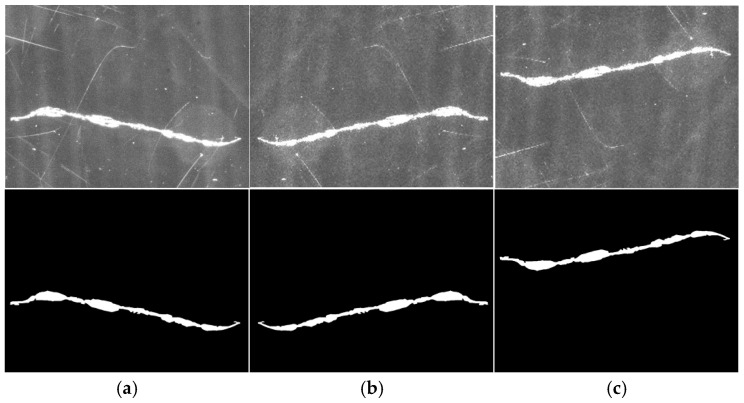
Image flip. (**a**) Defect original image, (**b**) flip horizontal, (**c**) flip vertical.

To enhance the generalization capability of deep learning models, image mirroring techniques are employed to augment training data. This study primarily applies horizontal and vertical flips to defect images, with horizontal flipping centered on the horizontal axis and vertical flipping centered on the vertical axis. To ensure consistency between defect images and their corresponding labeled images during augmentation, identical flipping operations are synchronized for both filtered defect images and labeled images. The results of this synchronized flipping method are illustrated in Figure 12. This approach not only enhances dataset diversity but also ensures precise alignment between defect images and their labels during model training, thereby improving defect detection accuracy.

b image translation

**Figure 13:**
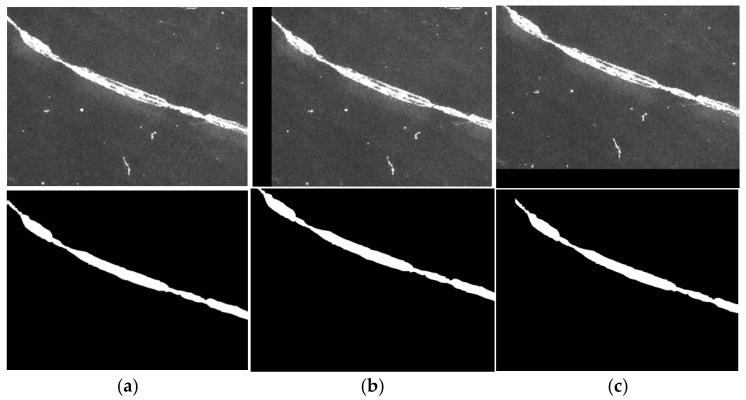
Image translation. (**a**) Defect original image, (**b**) vertical translation, (**c**) horizontal translation.

Image translation is a widely used data augmentation technique that generates new samples by shifting images horizontally or vertically by predefined distances. This method not only avoids the information loss caused by scaling but also significantly enhances training dataset diversity. The same translation process is applied simultaneously to both filtered defect images and their corresponding label maps. The translated defect images are illustrated in Figure 13.

c picture orientation

**Figure 14:**
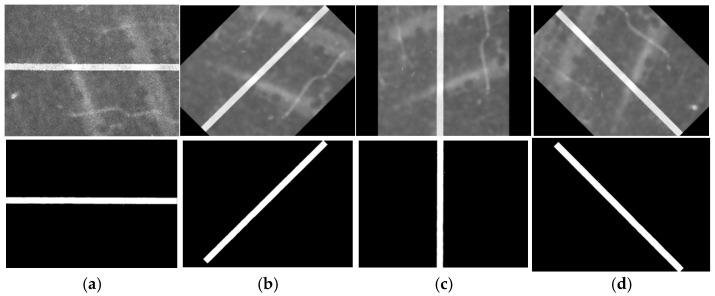
Image rotation. (**a**) Defect original image, (**b**) rotated 45°, (**c**) rotated 90°, (**d**) rotated 135°.

To preserve the original defect features in the dataset, the filtered surface defect images were rotated around a central point using three selected angles: 45°, 90° and 135°. These rotation angles effectively retain the original defect characteristics while generating new sample images with different orientations, thereby enhancing the diversity of the defect dataset. Identical rotation operations were applied to both images and their labels to ensure directional consistency and prevent annotation bias. Figure 14 demonstrates the results of the rotated images.

**Figure 15:**
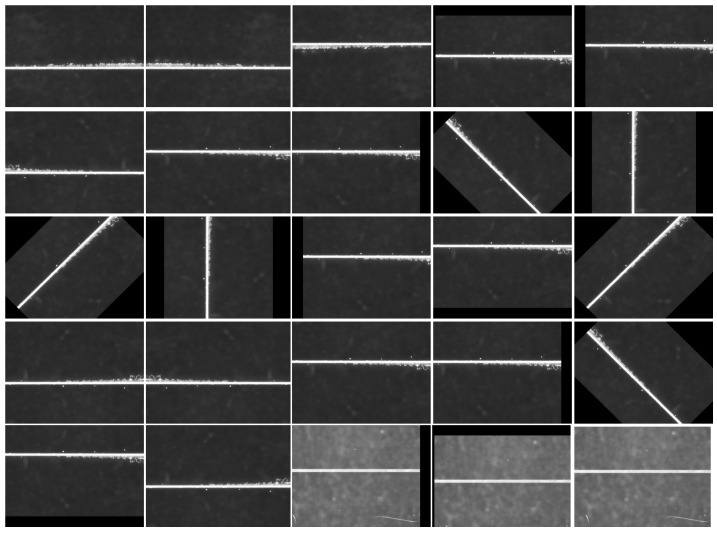
Surface defect image after data enhancement.

**Figure 16:**
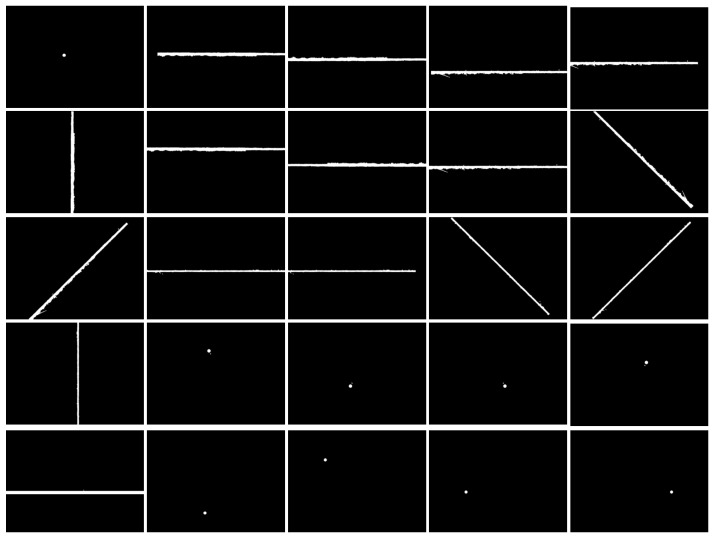
Surface defect label image after data enhancement.

After data augmentation, the dataset contains 4380 defect samples, including 2100 speckle defects and 2280 scratch defects. A subset of the surface defect images after data augmentation is shown in Figure 15 and Figure 16.

To evaluate model performance, the augmented defect images were divided into two parts: a training set and a test set. The training set serves as input data for network training to achieve model parameter optimization, while the test set evaluates the model’s generalization ability and detection accuracy. In this study, 4380 optical component surface defect images were processed through data augmentation, each with a resolution of 1280 × 720 pixels. Using the 4:1 hold-out ratio the dataset was partitioned into 3066 training images and 1314 test images. This division ensures balanced training and testing for the network model, providing a reliable foundation for accurate performance evaluation.

### 4.2. Evaluating Indicator

Accurate and comprehensive evaluation of the target recognition model’s performance is essential for assessing its effectiveness. In this paper, four metrics—mAP, precision, recall and parameter quantity—are employed to validate the algorithm’s performance in target recognition tasks. The calculation formulas for precision and recall are as follows:

(1) $$precision=\frac{TP}{TP+FP}$$

(2) $$recall=\frac{TP}{TP+FN}$$

In this formula, TP (true positives) represents correctly predicted positive cases (actual positive and predicted positive); FN (false negatives) represents missed positive cases (actual positive but predicted negative); FP (false positives) represents incorrectly predicted positive cases (actual negative but predicted positive); and TN (true negatives) represents correctly predicted negative cases (actual negative and predicted negative).

### 4.3. Experimental Results and Analysis

In this paper, the most widely used YOLO series models and the proposed BRCG-YOLOv8 model were trained for 200 epochs on the LLVIP dataset. The input image size was 640 × 640 pixels, with a batch size of four and an initial learning rate of 0.001. The experimental environment is detailed in Table 4.

#### 4.3.1. Experimental Environment Setup

Equipment and environment is shown in Table 4.

**Table 4:** Experimental environment.

Computing Platform	PC
CPU	Intel core i9-9900k CPU @3.60 GHz
GPU	NVIDIA RTX3090*2
internal storage	64 G
memory	11 GB GDDR6
software environment	Windows 10 Professional, Python3.6.2, CUDA11.6

#### 4.3.2. Analysis of Network Training Results

**Figure 17:**
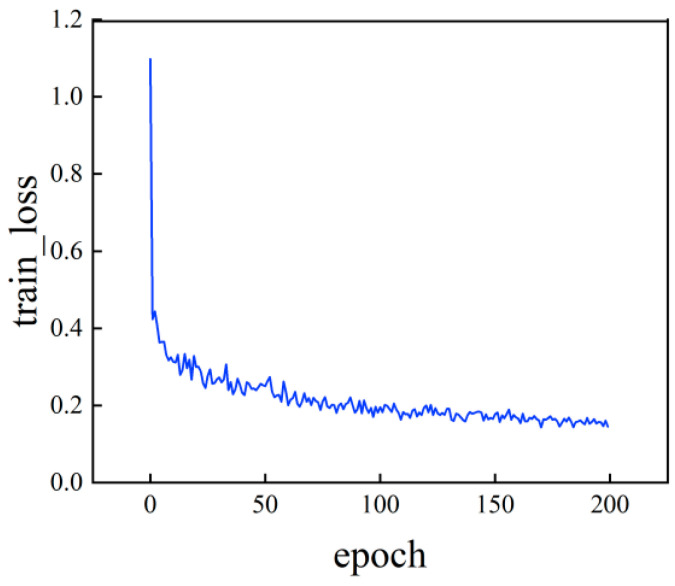
Loss curve variation.

Building upon the previously established dataset of surface defect optical components, systematic training experiments were conducted on the improved YOLOv8 network model. To enhance stability and convergence during initial training phases, the Adam optimizer with dynamic parameter adjustments was employed, initializing the learning rate to zero. Through 200 iterations of iterative optimization using microscopic dark-field defect images, the network’s ability to recognize surface defect features was progressively enhanced. Figure 17 illustrates the loss function variation curve of this network model during training.

As shown in Figure 17, the network training loss demonstrates a downward trend with increasing iterations. The loss value decreases progressively until reaching relative stability, reflecting the network’s continuous performance optimization during learning. Notably, the loss experienced a sharp decline during the first 50 iterations, highlighting the model’s rapid learning capability in its early stages. When iterations reached 200, the rate of loss reduction slowed significantly, eventually stabilizing around 0.18. This indicates that the network has achieved convergence, completing its training process and approaching optimal performance. The trained model was then applied to a dark-field image dataset using the enhanced YOLOv8 network for performance analysis.

**Figure 18:**
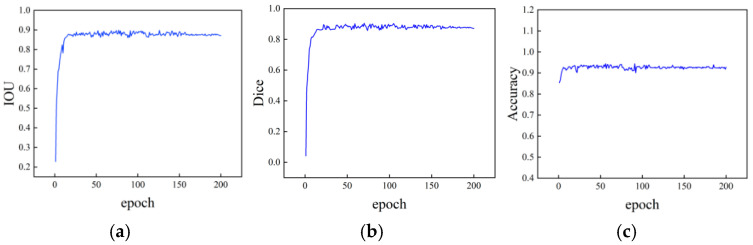
Curve variation in different evaluation indexes. (**a**) Compare and cross, (**b**) semblance, (**c**) accuracy rate.

As shown in Figure 18, after the network model stabilized during training, the intersection-over-union ratio and similarity coefficient reached approximately 0.87, with an accuracy rate of about 0.92. These results indicate that, while the network can partially identify surface defects on optical components, a noticeable gap remains between the generated detection results and the true labels, suggesting that detection performance has not yet reached ideal levels. To further improve the quantitative analysis accuracy of surface defects in optical components, optimizing the network architecture or adjusting training strategies is necessary to enhance the feature extraction precision of YOLOv8 for surface defect detection.

#### 4.3.3. Ablation Experiment

To verify the effectiveness of every module, the ablation experiments are conducted in the study. The performance improvement of the hybrid attention mechanism and efficient feature convolution module were evaluated (Table 5) by comparing the performance of the original network, the ablation model and the final network. The experimental results demonstrate that the newly added modules significantly improved the repeatability and positioning accuracy of feature point extraction.

According to Table 5, the network with the embedded hybrid attention module achieved an average improvement of 1.3% in feature point repeatability and 6.2% in the single correspondence threshold 1 accuracy. The convolution module network of efficient features improved by 1.8% and 5.6%, respectively. The improvement in repeatability indicates that more correct feature points were obtained, but the positioning error of the single module network decreased due to the introduction of high-precision feature points combined with distant outliers. After integrating the two modules into the algorithm, the three indicators (repeatability, positioning error and average accuracy) improved by 2.6%, 0.9% and 8.2% compared to the original network. The optimization of the positioning error originated from the increase in high-accuracy feature points and the reduction in distant outliers. Ablation experiments verified the effectiveness of the modules and the performance advantages of the network in this study. In terms of model lightweighting, the hybrid attention module had negligible impact on the number of parameters and computational cost. After introducing the efficient feature convolution module, the computational cost and parameter count were reduced to 37.7% and 43.7% of the original network, indicating that the algorithm significantly reduces redundancy while maintaining performance.

#### 4.3.4. The Results and Analysis of the Surface Defect Extraction

**Figure 19:**
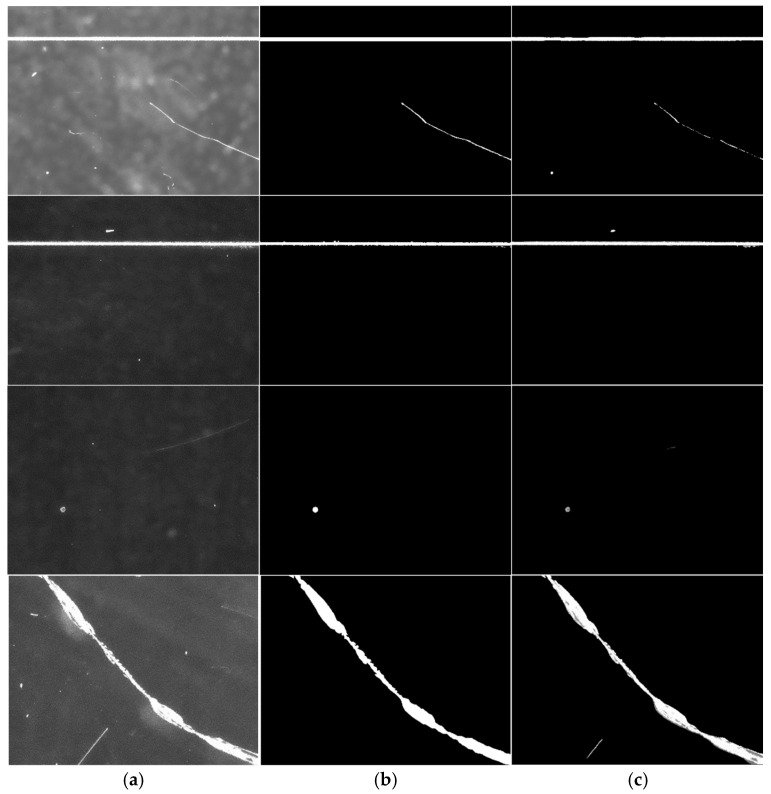
Dark defect image detection results. (**a**) Defect original image. (**b**) Label Image. (**c**). Inspection result image.

By selecting the optimal weights obtained from training the improved YOLOv8 network model, rapid defect prediction was achieved. The target defects were extracted from dark-field images in just 0.02 s, as shown in Figure 19.

By comparing the predicted results with their labels, it is clearly observable that the method can effectively detect target scratches and spots in surface defect images. However, the detection results still exhibit significant noise interference, with some background noise misidentified as target defects. Additionally, the edges of segmented target defects are not sufficiently clear, showing blurring or breakage phenomena. These issues hinder the pixel-based defect information extraction method from accurately calculating the specific shape, size and position of defects, thereby affecting the accuracy of quantitative analysis and quality assessment. Therefore, the network model requires further improvements to reduce non-target noise detection and enhance edge extraction accuracy for target defects.

## 5. Conclusions

Based on the characteristics of surface defects of optical components, a novel YOLOv8 target recognition algorithm was proposed in the paper. The main contributions focus on the following three aspects. Firstly, the microscopic dark-field imaging technology is employed to collect defect images and the collected images are processed to construct a surface defect dataset.

Secondly, defect features are analyzed under microscopic dark-field conditions. An improved YOLOv8 network is designed to extract surface defects from the components. The architecture of the improved YOLOv8 and the parameter settings during model training are meticulously optimized. Thirdly, the constructed defect dataset is used to train the improved YOLOv8t network model, and the performance of the trained model is evaluated using similarity coefficient, intersection-over-unio and accuracy rate. The results of the ablation experiments show that the network with the embedded hybrid attention module achieves an average improvement of 1.3% in feature point repeatability and 6.2% in single correspondence threshold 1 accuracy compared to the original network. The efficient feature convolution module contributes an improvement of 1.8% and 5.6%, respectively, in the same metrics. After integrating both modules, the algorithm’s performance in repeatability, positioning error and average accuracy rate improves by 2.6%, 0.9% and 8.2%, respectively. The optimization of the positioning error arises from the increase in high-accuracy feature points and the reduction in distant outliers. Ablation experiments verify the effectiveness of the modules and the performance advantages of the proposed network. The experimental results indicate that the improved YOLOv8 network can effectively detect surface defect regions but is still affected by non-target noise points, and the edges of extracted target defects remain unclear. Consequently, the network model requires further improvements to enhance the accuracy of surface defect extraction and reduce the impact of noise on detection results.

## Figures and Tables

**Table 1:** Typical surface defect type.

Defect Type	Dig	Scratch	Broken Edge	Bubble
Appearance	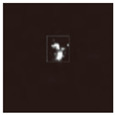	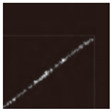 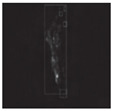	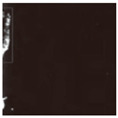	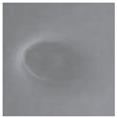
ISO 10110-7	Length < 2 mm	Long scratch length > 2 mm	Description requirements to be met	Gas not discharged in time during production and processing
GB/T 1185-2006	Rough pockmarks corresponding to basic series of tolerance	Aspect ratio of long scratch > 160:1		
MIL-PRF-13830 B	A maximum size pockmark is allowed in the 20 mm area	Maximum size–total scratch length < One quarter of the element diameter	Ignored when not entering the effective aperture	One is allowed in the area with an optical diameter of 20 mm

**Table 2:** The comparison results of surface defect detection among main optical elements.

Test Method	Core Principles	Advantage	Limitations	Typical Accuracy/Applications
Dark-field scattering method	Defects cause light scattering	Sensitive to small defects, the equipment is relatively simple	Small depth-to-width ratio/smooth defects may be missed	Submicron level
Microscopic dark-field + reverse identification	Scattering imaging + electromagnetic simulation + inverse solving	Quantifiable 3D information with high accuracy	The system is complex, the calibration requirement is high, and the speed is relatively slow	<100 nm
Deep UV interference method	193 nm interferometric wavefront measurement	Ultra-high resolution, comprehensive evaluation of surface shape and local defects	The equipment is expensive and the environment is demanding	At the nanoscale, wafer/high-end objective lens
Focus on 3D reconstruction	Point scan optical tomography + 3D modeling	It provides true 3D morphology and strong chromatographic capacity	Slow scanning speed, high equipment cost	Submicron, VR lens
Deep learning	Image acquisition + AI feature learning and classification	High automation, adapt to complex texture, fast speed, standard unified	The model needs to be optimized/updated by relying on a large amount of annotated data	Meet the needs of production line, improve efficiency and reduce cost
Blazed compression scan	Prism compresses light spot + scan image	Improving the resolution of a single dimension is suitable for detecting specific structures	Application scenarios are relatively specific	Wafer defects

**Table 3:** Main optical parameters of zoom lens.

Lens Model	SPZF0763 + ZF-1.42C		
optical magnification	1.0×~9.0×		
operating distance	90 mm		
multiplying power	1.0×	3.0×	9.0×
NA	0.02	0.048	0.08
resolution ratio (μm)	16.8	7.0	4.2
depth of field (mm)	2.0	0.27	0.05
aberration	0.21%	0.19%	0.14%
maximum compatible camera	2/3″CCD		
install	C joggle		

**Table 5:** Comparison of ablation experiment results.

Algorithm	Rep ↑	Localization Error ↓	Correctness ↑	FLOPs/G ↓	Params ↓
SuperPoint	0.612	1.078	0.681	26.289	1.304
SuperPoint-CBAM	0.620	1.095	0.723	26.290	1.306
SuperPoint-Efficient Feature Convolution	0.623	1.094	0.719	9.899	0.568
Ours algorithm	0.628	1.068	0.737	9.900	0.570

↑ = The larger the better; ↓ = The smaller the better.

## Data Availability

The original contributions presented in this study are included in the article. Further inquiries can be directed to the corresponding author.
